# Development and validation of a risk prediction model for radiotherapy-related esophageal fistula in esophageal cancer

**DOI:** 10.1186/s13014-019-1385-y

**Published:** 2019-10-22

**Authors:** Yiyue Xu, Linlin Wang, Bo He, Wanlong Li, Qiang Wen, Shijiang Wang, Xindong Sun, Xue Meng, Jinming Yu

**Affiliations:** 1 School of Medicine, Shandong University, Jinan, China; 2 Department of Radiation Oncology, Shandong Cancer Hospital and Institute, Shandong First Medical University and Shandong Academy of Medical Sciences, No.440, Ji Yan Road, Jinan, Shandong 250012 People’s Republic of China; 3 Provincial Hospital Affiliated to Shandong University, Jinan, China

**Keywords:** Esophageal cancer, Radiotherapy, Esophageal fistula, Risk factors, Nomogram

## Abstract

**Objectives:**

We aimed to identify the risk factors and provide a nomogram for the prediction of radiotherapy-related esophageal fistula in patients with esophageal cancer (EC) using a case-control study.

**Patients and methods:**

Patients with esophageal fistula who received radiotherapy or chemoradiotherapy between 2003 and 2017 were retrospectively collected in two institutions. In the training cohort (TC), clinical, pathologic, and serum data of 136 patients (cases) who developed esophageal fistula during or after radiotherapy were enrolled and compared with 272 controls (1:2 matched with the diagnosis time of EC, sex, marriage, and race). After the univariable and multivariable logistic regression analyses, the independent risk factors were identified and incorporated into a nomogram. Then the nomogram for the risk prediction was externally validated in the validation cohort (VC; 47 cases and 94 controls) using bootstrap resampling.

**Results:**

Multivariable analyses demonstrated that ECOG PS, BMI, T4, N2/3 and re-radiotherapy were independent factors for esophageal fistula. Then a nomogram was constructed with the C-index of 0.805 (95% CI, 0.762–0.848) for predicting the risk of developing esophageal fistula in EC patients receiving radiotherapy. Importantly, the C-index maintained 0.764 (95% CI, 0.683–0.845) after the external validation.

**Conclusions:**

We created and externally validated the first risk nomogram of esophageal fistula associated with radiotherapy. This will aid individual risk stratification of patients with EC developing esophageal fistula.

## Introduction

Esophageal cancer is a common malignancy of the digestive tract, ranking the 8th in global malignant tumors [1]. The incidence of EC in China is higher than the global average and is the fourth leading cause of cancer-related death [2]. Radiotherapy is one of the main therapeutic strategies for the control of EC disease, which includes the neoadjuvant/adjuvant therapy, radical radiotherapy and palliative radiotherapy [3]. Nearly one half of the EC patients will receive radiotherapy during their disease course [4–6]. However, the adverse events unavoidably occurred, and sometimes threatened the patient life.

Esophageal fistula is one of the serious complications related with the radiotherapy, and 4.3–24% patients were reported to develop esophageal fistula after the chemoradiotherapy [4, 7–13]. Due to the related infection, massive hemorrhage or unhealed abscess [14–17], the prognosis of patients with esophageal fistula was extremely poor; with the median survival of only two or 3 months [17–19]. Therefore, early prediction of esophageal fistula is very important for reducing the risk of death and improving the patient prognosis.

Some studies have shown that low serum cholesterol level, T4 stage, ulcerative type, and re-radiotherapy were related to the occurrence of esophageal fistula [12, 15–17, 19, 20]. However, the sample size is always relatively small, and often limitedly enrolled patients with advanced squamous cell carcinoma [16]. Besides, some patients with the esophageal fistula also received the treatment of surgery which may also contribute to the fistula [21]. Thus, the assessment of risk from radiotherapy may be influenced by the confound factors. More importantly, no risk model has been established for quantitatively predicting the occurrence of esophageal fistula.

Therefore, we attempted to retrospectively analyze the risk factors of esophageal fistula in EC patients who received radiotherapy but not surgery, and developed a nomogram to predict the occurrence of radiotherapy-related esophageal fistula.

## Materials and methods

### Patients

This retrospective study was approved by the Institutional Review Board of Shandong Cancer Hospital and Shandong Provincial Hospital. For this retrospective study, formal written informed consent from all patients was not required, and all data were kept confidential. The patient records of all EC patients treated with radiotherapy or chemoradiotherapy between 2003 and 2017 were reviewed and the patients who developed esophageal fistula were further identified. The inclusion criteria were 1) diagnosed as EC by pathological biopsy; 2) received radical radiotherapy/chemoradiation, palliative radiotherapy/chemoradiation, or a second course of radiotherapy to EC; 3) diagnosed as esophageal fistula by endoscopy, CT or X-ray with meglumine diatrizoate. Exclusion criteria were 1) underwent esophageal surgery; 2) other causes contributed to the esophageal fistula, such as medical injure or trauma; 3) the fistula was developed before treatment or assessed by the disease progression; 4) concomitant with another carcinoma. Eligible patients who developed esophageal fistula were included into the case group. Controls were matched to cases with 1:2 by the diagnosis time of EC, sex, marriage, and race. One hundred thirty-six patients with esophageal fistula and 272 controls in Shandong Cancer Hospital were used as training cohort (TC); 47 cases and 94 controls from Shandong Provincial Hospital were separately assigned as the independent external validation cohort (VC).

### Data collection

Data were collected using a standardized questionnaire. Information collected included general (age, ECOG PS, BMI, history of smoking and history of diabetes), diagnostic (T4, N2/3, longitudinal length of lesions and general type), treatment-related (re-radiotherapy, radiotherapy dose and chemotherapy), and hematological data (serum cholesterol and albumin) which is reported in Additional file 1. All the data were 1 month before radiotherapy.

### Variables definition

ECOG PS is defined as Eastern Cooperative Oncology Group performance status. BMI (Body Mass Index) is calculated using the international standard method: weight squared/height. Smoking is defined as smoking at least one cigarette a day for at least 1 year. TNM stage is in accordance with the UICC-TNM classification 7th edition. Chemotherapy is defined as receiving chemotherapy before radiotherapy or concurrent chemoradiotherapy.

### Statistical analysis

The association of various factors with the risk of esophageal fistula were assessed by conditional logistic regression. 95% confidence intervals (95% CI) and *P* value are calculated for overall effect for factors in the study. Based on a *P* value < 0.1 in univariate analyses, factors were selected into the multivariate analysis. The nomogram for the prediction of probability of radiotherapy-related esophageal fistula were established with the results of multivariate analysis.

The predictive ability of the nomogram was assessed by concordance index (C-index). Calibration curves were analyzed by plotting the nomogram predicted and the actual probability of the occurrence of esophageal fistula. During the external validation, the total points of each patient in the validation cohort were calculated according to the established nomogram, then logistic regression in this cohort was performed using the total points as a factor, and finally, the C-index and calibration curve were derived based on the regression analysis.

Statistics analysis were performed using SPSS 24.0 version and R version 3.5 for Windows.

## Results

### Patient characteristics

**Fig. 1:**
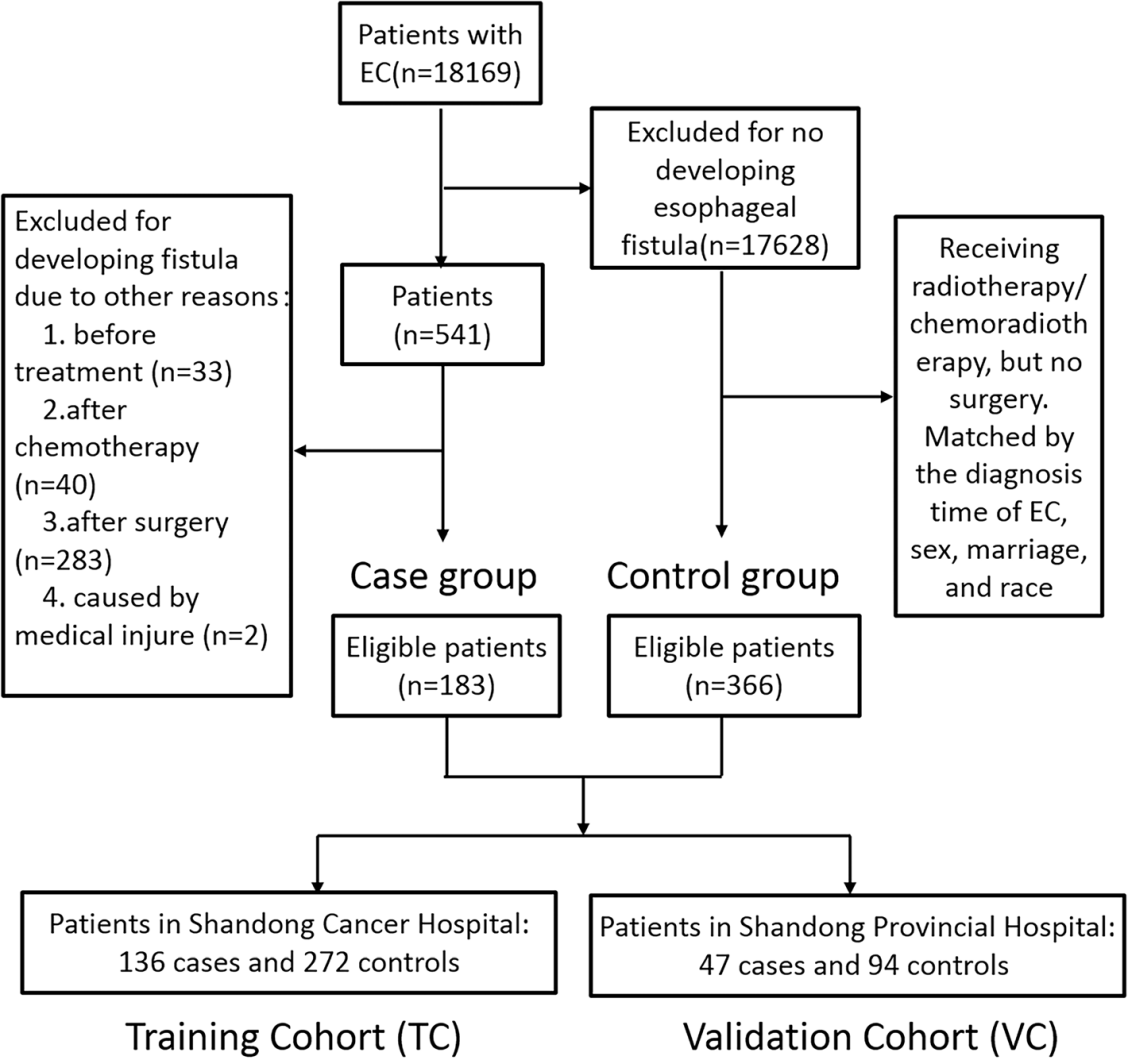
Flow chart for patient selection

During the study period, 18,169 EC patients were treated with radiotherapy in these two institutions. Esophageal fistula occurred in 541 patients. After excluding patients who developed fistula due to other reasons, 183(1.01%) EC patients developed esophageal fistula during or after radiotherapy. Controls who received radiotherapy/chemoradiotherapy but no surgery were matched to cases. All eligible patients were divided into TC and VC by different treatment institutions (Fig. 1).

Among the 183 EC patients, 88 were diagnosed as fistula by CT; 84 were diagnosed by esophagogram; 11 were diagnosed by endoscopic. Perforation occurred during radiotherapy in 38 patients, while 145 patients developed fistula after radiotherapy. All patients who developed esophageal fistula during radiotherapy discontinued radiotherapy. The median intervals between the termination of radiotherapy and occurrence of fistula were 5 months. In 145 patients who finished the radiotherapy, a majority of patients (97) accepted a dose ≥60Gy; 36 patients accepted ≥50 < 60Gy; and only 12 patients accepted <50Gy. In the treatment methods of esophageal fistula, 39 patients were treated conservatively with parenteral nutrition, 61 with esophageal stent, 41 with nutrient canal, 17 with gastrostomy, 2 with radical resection (Table 1).

**Table 1:** Characteristics of patients with radiotherapy-related esophageal fistula

Characteristics	Training cohort		Validation cohort	
	N	%	N	%
Age (years), mean ± SD	61.8 ± 8.9		60.2 ± 10.7	
ECOG PS				
≤ 2	94	69.1	32	68.1
≥ 3	42	30.9	15	31.9
T stage				
T1–3	85	62.5	34	72.3
T4	51	37.5	13	27.7
N stage				
N0–1	69	50.7	20	42.6
N2–3	67	49.3	27	57.4
Stage				
I	0	0	0	0
II	14	10.3	5	10.6
III	91	66.9	29	61.7
IV	31	22.8	13	27.7
Chemotherapy				
Yes	98	72.1	33	70.2
No	38	27.9	14	29.8
Re-radiotherapy				
Yes	25	18.4	6	12.8
No	111	81.6	41	87.2
Single dose of radiation (Gy)				
≤ 2	129	4.9	7	0.0
> 2	7	5.1	0	0
Total dose (patients who finished the radiotherapy)				
≥ 60Gy	58	57.4	39	88.6
≥ 50 < 60Gy	34	33.7	2	4.6
< 50Gy	9	8.9	3	6.8
Diagnostic tool				
CT	76	55.9	12	25.5
Esophagogram	59	43.4	25	53.2
Endoscopic	1	0.7	10	21.3
Occurrence time				
During radiotherapy	35	25.7	3	6.4
After radiotherapy	101	74.3	44	93.6
Median time between the end of radiotherapy and fistula (month)	4.5		5.5	
Fistula type				
Esophageal-respiratory	67	49.3	40	85.1
Esophageal-mediastinum	66	48.5	4	8.5
Esophagopleural fistula	2	1.5	1	2.1
Both esophageal-respiratory and esophageal-mediastinum fistula.	1	0.7	2	4.3
Therapy				
Conservative treatment	31	22.8	8	17.0
Esophageal stent	31	22.8	30	63.8
Nutrient canal	36	26.5	5	10.6
Gastrostomy	15	11.0	2	4.3
Radical resection	2	1.5	0	0
Others	21	15.4	2	4.3

### Univariate analysis and multivariate analysis of risk factors associated with esophageal cancer

In the TC, conditional logistic regression was performed for the determination of the potential risk factors of esophageal fistula. On the univariable logistical regression analysis, a significant difference can be detected between the cases and controls in terms of ECOG PS ≥ 3, BMI, T4, N2/3, the longitudinal length of lesions, re-radiotherapy, Taxol chemotherapy and serum cholesterol (p all < 0.1). No significant differences were observed regarding smoking history, diabetes history, and other factors.

On multivariate analysis, ECOG PS ≤ 2 and BMI ≥ 18.5 kg/ m^2^ were the protective factors for the occurrence of esophageal fistula. On the contrary, T4, N2/3 and re-radiotherapy were the independent risk factors for EC patients (Table 2).

**Table 2:** Univariate and multivariate analysis of risk factors in training cohort

Factors	Univariate		Multivariate	
	P	OR (95.0% CI)	P	OR (95.0% CI)
Age (years)				
< 60	0.944	1.015 (0.673–1.529)		
≥ 60				
ECOG PS				
≤ 2	< 0.001	7.922 (3.828–16.396)	< 0.001	5.165 (2.180–12.242)
≥ 3				
BMI (kg/m^2^)				
< 18.5	< 0.001	1.00(reference)	0.001	1.00(reference)
18.5–23.9		0.283 (0.138–0.583)		0.371 (0.164–0.837)
24–27.9		0.154 (0.066–0.360)		0.176 (0.065–0.480)
≥ 28		0.038 (0.010–0.150)		0.059 (0.012–0.287)
History of Smoking				
No	0.137	1.381 (0.903–2.112)		
Yes				
History of diabetes				
No	0.638	0.843 (0.414–1.716)		
Yes				
T stage				
T1–3	< 0.001	2.853 (1.763–4.617)	0.018	2.123 (1.137–3.965)
T4				
N stage				
N0–1	< 0.001	2.355 (1.527–3.629)	0.003	2.489 (1.377–4.499)
N2–3				
Longitudinal length of lesions	0.020	1.096 (1.015–1.185)	0.246	1.064 (0.958–1.181)
General type				
Medullary type	0.409	1.00(reference)		
Mushroom type		1.458 (0.810–2.627)		
Ulcerative type		1.607 (0.936–2.760)		
Constrictive type		0.992 (0.448–2.196)		
Cavity type		0.941 (0.278–3.182)		
Re-radiotherapy				
No	< 0.001	6.262 (2.682–14.620)	< 0.001	10.392 (3.491–30.938)
Yes				
Single dose of radiation (Gy)				
≤ 2	1.000	1.000 (0.404–2.478)		
> 2				
Chemotherapy				
No	0.500	1.166 (0.746–1.821)		
Yes				
Chemotherapy				
0 line	0.307	1.00(reference)		
1 line		1.072 (0.678–1.695)		–
2 line		1.990 (0.841–4.710)		–
3 line and more		2.367 (0.496–11.306)		
Taxol chemotherapy				
No	0.031	1.592 (1.043–2.428)	0.128	1.524 (0.886–2.622)
Yes				
Serum cholesterol (mmol/l)				
< 4.40	0.029	0.819 (0.656–1.024)	0.056	0.557 (0.305–1.016)
≥ 4.40				
Serum albumin (g/dl)				
< 3.5	0.495	0.936 (0.887–0.988)		
≥ 3.5				

### Prognostic nomogram and the validation of predictive accuracy

**Fig. 2:**
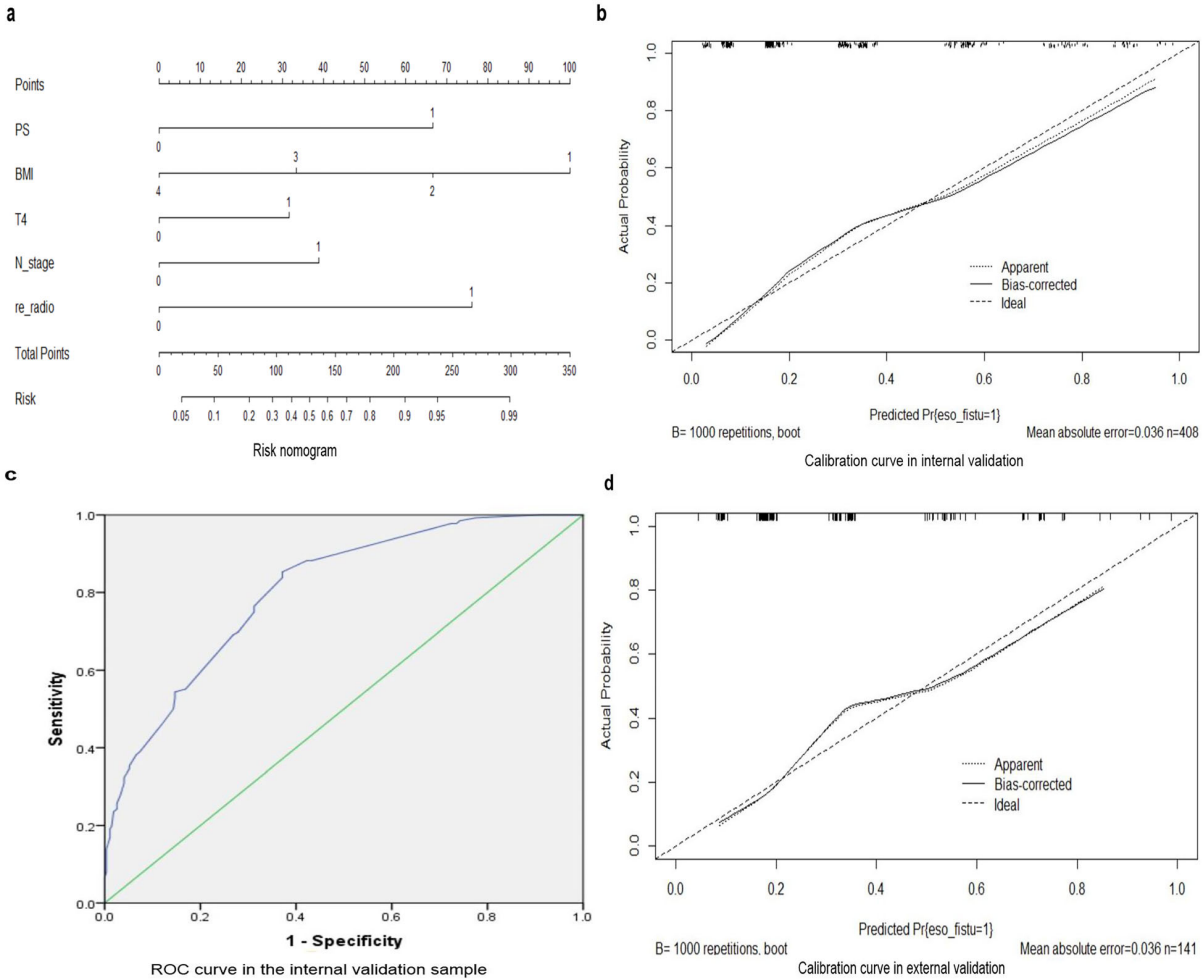
**a** Nomogram for individualized prediction of radiotherapy-related esophageal fistula in patients with esophageal cancer. PS 0: ECOG PS ≤ 2; 1: ECOG PS ≥3. BMI 1: < 18.5 kg/m^2^; 2: 18.5–23.9 kg/m^2^; 3: 24–27.9 kg/m^2^; 4: ≥28 kg/m^2^. T4 0: T1–3 stage; 1: T4 stage. N_stage 0: N0–1 stage; 1: N2–3 stage. re_radio 0: no re-radiotherapy; 1: re-radiotherapy. **b** Calibration curve in internal validation. **c** Receiver under the operator characteristic (ROC) curve for the test accuracy of the final risk score in the internal validation sample (C-index = 0.805, 95% CI 0.762 to 0.848). **d** Calibration curve in external validation

Based on these independent factors, a nomogram was constructed for predicting the probability of the occurrence of esophageal fistula in EC patients receiving radiotherapy. The point of each factor can be determined according to the intersection of the vertical line drawn from the variable to the point axis. Then, the total risk score was calculated by adding all the variable points; and the probability of the occurrence of esophageal fistula can be directly read on the total point axis. The C- index of this risk nomogram was 0.805, (95% CI: 0.762–0.848). The calibration curves showed a good agreement between the risk estimation by the nomogram and actual observation (Fig. 2).

The nomogram’s performance was then assessed in the VC. A C-index of 0.764 (95% CI: 0.683–0.845) was observed. The calibration curves also indicated that the predicted probabilities by the nomogram were good match with the clinical confirmation (Fig. 2).

## Discussion

This study comprehensively evaluated the risk factors in the occurrence of radiotherapy-related esophageal fistula in patients with EC and developed a reliable nomogram. Of the currently available prediction tools, a nomogram is a graphic representation of the solution of an equation and has a good discrimination characteristic in predicting outcomes which is easy to use [22]. To our best knowledge, the established nomogram is the first model for predicting esophageal fistula probability and externally validated.

In the present study, patients with T4 stage had higher probability to develop esophageal fistula, due to the deeper tumor invasion depth. When tumor declined after radiotherapy, normal tissue repaired relatively slowly, which was easy to develop fistula [20, 23]. Consistent with the findings of conformal radiotherapy by Chen Haiyan [19], we found that the proportion of patients with T4 disease in the case group was much higher than that in the control group (35.0% vs. 16.9%). Besides, N2/3 was another independent risk factor. The increase of N staging lead to the enlargement of the target area according to the NCCN Guidelines which are much more likely to increase the risk of developing esophageal fistula.

Our study also suggested re-radiotherapy would increase the risk of radiation-related esophageal fistula; it may demonstrate that patients with T4 or N2/3 should be more cautious in the choice of re-radiotherapy to avoid esophageal perforation [24]. The risk score can help to stratify the patients with high risk of radiation-related esophageal fistula and provide alternative therapeutic strategy other than re-radiotherapy for local recurrence, such as surgery and chemotherapy.

Tsushima [16] reported the effect of BMI on the occurrence of esophageal fistula, but only compared the difference between BMI ≤ 20 and BMI > 20. In this study, BMI was divided to four grades according the international standard, so that the differences between different BMI could be compared more accurately. BMI was found to be a protective factor for the esophageal fistula; less BMI was associated with the more elevated risk of esophageal fistula. Moreover, the patients with high ECOG PS also was the independent factor for the developing of esophageal fistula. High ECOG PS and low BMI were related to the poor nutritional [25] and immune status [26], which may cause the impaired capability of repairing in tumor tissues.

Although the level of serum cholesterol was found to be related with the occurrence of esophageal fistula, patients with less than 170 mg/dl were 14.7 times more likely to develop arterio-esophageal fistula after chemoradiotherapy than those with high cholesterol; only 48 samples were included in the study [15]. In our present study, the patients in the esophageal fistula group also had lower total cholesterol level in univariate logistic regression analysis, but lose the statistical significance in multivariate, demonstrating the level of serum cholesterol may be influenced by other risk factors and was a confounding factor.

Using the nomogram, patients with high scores (> 180 points) are 80% more likely to develop esophageal fistula. Clinicians should ensure that esophagogram is examined once a week during radiotherapy and pay more attention on these patients during follow-up. Timely treatment should be given to patients who cough or choke drinking water. Studies have shown that esophageal bypass operation before definitive chemoradiotherapy can reduce the incidence of esophageal fistula [27].

This study has several limitations. As a case control study, there may be selection bias. We use a third party to collect data in both case and control group, to minimize observational bias. In addition, this study was a retrospective study in which some patients were enrolled without endoscopic ultrasonography, so the parameters of esophageal stenosis can’t be assessed. Because the study population was all Han Chinese, the results may be verified in the western patients and the prospective clinical trials were urgently needed in the future.

## Conclusion

Combing the risk factors, a nomogram was constructed and externally validated for the prediction of esophageal fistula associated with radiotherapy. This tool might be helpful for individualized stratifying the patients with different risk of esophageal fistula and lead to a rational therapeutic choice in patients with EC.

## Supplementary information

**Additional file 1.** Characteristics of patients.

## Data Availability

The datasets used and/or analyzed during the study are available from the corresponding author on reasonable request.

## Abbreviations

BMI: Body mass index
CI: Confidence interval
C-index: Concordance index
EC: Esophageal cancer
ECOG PS: Eastern Cooperative Oncology Group performance status
TC: Training cohort
TNM: Tumor-node-metastasis
VC: Validation cohort
