# Supplementary material for: Development and validation of a risk prediction model for radiotherapy-related esophageal fistula in esophageal cancer
Source: Radiat Oncol. 2019 Oct 22;14:181. doi: 10.1186/s13014-019-1385-y (PMC6805370; doi:10.1186/s13014-019-1385-y)
Supplement: Supplementary file 1 — Additional file 1. Characteristics of patients. [file 13014_2019_1385_MOESM1_ESM.docx]

**Additional file1 Characteristics of patients**

| **Factors** | **Case group** | |  | **Control group** | |
| --- | --- | --- | --- | --- | --- |
|  | **N** | **%** |  | **N** | **%** |
| **Age (years)**  <60  ≥60 | 84  99 | 45.9  54.1 |  | 166  200 | 45.4  54.6 |
| **ECOG PS**  ≤2  ≥3 | 126  57 | 68.9 31.1 |  | 339 27 | 92.6 7.4 |
| **BMI (kg/m²)**  <18.5  18.5-23.9  24-27.9  ≥28 | 40 112 28 3 | 21.9 61.2 15.3 1.6 |  | 25 200 95 46 | 6.8 54.6 26.0 12.6 |
| **History of Smoking**  no  yes | 68  115 | 37.2  62.8 |  | 147  219 | 40.2  59.8 |
| **History of diabetes**  no  yes | 166  17 | 90.7  9.3 |  | 334  32 | 91.3  8.7 |
| **T stage**  T1-3  T4 | 119 64 | 65.0 35.0 |  | 304 62 | 83.1 16.9 |
| **N stage**  N0-1  N2-3 | 89 94 | 48.6 51.4 |  | 250 116 | 68.3 31.7 |
| **Longitudinal length of lesions (mean ± SD)** | 4.96±2.92 | |  | 5.14±2.45 | |
| **General type**  medullary type  mushroom type  ulcerative type  constrictive type  cavity type | 77 45 43 12 6 | 42.1 24.6 23.5 6.5 3.3 |  | 201 64 58 31 12 | 54.9 17.5 15.8 8.5 3.3 |
| **Re-radiotherapy**  no  yes | 152  31 | 83.1  16.9 |  | 352  14 | 96.2  3.8 |
| **Single dose of radiation (Gy)**  ≤2  >2 | 176 7 | 96.2 3.8 |  | 351 15 | 95.9 4.1 |
| **Chemotherapy**  no  yes | 52  131 | 28.4  71.6 |  | 109  257 | 29.8  70.2 |
| **Chemotherapy**  0 line  1 line  2 line  3 line and more | 52 114 12 5 | 28.4 62.3 6.6 2.7 |  | 109 235 18 4 | 29.8 64.2 4.9 1.1 |
| **Taxol chemotherapy**  no  yes | 89  94 | 48.6  51.4 |  | 212  154 | 57.9  42.1 |
| **Serum cholesterol (mmol/l)**  <4.40  ≥4.40 | 85  98 | 46.4  53.6 |  | 116  250 | 31.7  68.3 |
| **Serum albumin (g/dl)**  <3.5  ≥3.5 | 13  170 | 7.1  92.9 |  | 14  352 | 3.8  96.2 |
